# Complications and Outcomes of Surgically Treated Pediatric Supracondylar Humerus Fractures

**DOI:** 10.3390/children11070791

**Published:** 2024-06-28

**Authors:** Sebastian G. Hahn, Andrea Schuller, Lorenz Pichler, Anna Hohensteiner, Thomas Sator, Oskar Bamer, Britta Chocholka, Manuela Jaindl, Elisabeth Schwendenwein, Bikash Parajuli, Sanika Rapole, Thomas Tiefenboeck, Stephan Payr

**Affiliations:** 1 Department of Trauma Surgery, University Clinic of Orthopedics and Trauma Surgery, Medical University of Vienna, 1090 Vienna, Austria; sebastian.hahn@meduniwien.ac.at (S.G.H.); andrea.schuller@meduniwien.ac.at (A.S.); lorenz.pichler@meduniwien.ac.at (L.P.); anna.hohensteiner@meduniwien.ac.at (A.H.); thomas.sator@meduniwien.ac.at (T.S.); oskar.bamer@meduniwien.ac.at (O.B.); britta.chocholka@meduniwien.ac.at (B.C.); manuela.jaindl@meduniwien.ac.at (M.J.); elisabeth.schwendenwein@meduniwien.ac.at (E.S.); thomas.tiefenboeck@meduniwien.ac.at (T.T.); 2 Section of Pediatric Trauma Surgery, Department of Trauma Surgery, University Clinic of Orthopedics and Trauma Surgery, Medical University of Vienna, 1090 Vienna, Austria; 3 Department of Orthopedics and Traumatology, Dhulikhel Hospital, Kathmandu University Hospital, Kavre, Nepal; bikash@kusms.edu.np; 4 Department of Pediatric Orthopedics, Sancheti Institute for Orthopedics and Rehabilitation, Pune, India; drsanikarapole@gmail.com

**Keywords:** pediatric supracondylar humerus fracture, reduction, surgery, complications, outcome

## Abstract

This study describes the outcome of supracondylar humerus fractures in children using crossed K-wires after closed or open reduction with the medial, lateral or bilateral approach. Patients treated between January 2000 and December 2019 were classified according to the Von Laer classification, complications were classified according to the Sink classification and clinical outcomes were classified according to modified Flynn criteria. In total, 364 patients with a mean age of 5.23 ± 2.45 years were included. The majority were type IV fractures (156; 42.9%) and 94 (60.3%) needed an open reduction for which the medial approach (53; 56.4%) was predominantly used. Overall, of 50 complications (31 using closed reduction, 19 open reduction), 17/50 (34%) needed revision surgery. An excellent clinical outcome was achieved in 348/364 (95.6%) patients. The approach used for open reduction as such had no influence on the complication rate or clinical outcome. For severely displaced fractures, the data showed that an open approach for crossed K-wires tended to result in fewer complications and better clinical outcomes than a closed reduction. If an open reduction is indicated, the required approach (medial, lateral or bilateral) should be primarily selected according to the requirements of the fracture pattern and eventual cosmetic considerations.

## 1. Introduction

The supracondylar humerus fracture (SHF) is a common injury with an incidence of up to 14% of all fractures in childhood and adolescence and is increasing in boys and girls [1,2,3,4]. In children, this injury represents 48–78% of all fractures in the elbow joint [2,5,6].

The completion of the growth process of the pediatric elbow joint, which takes until the age of 19, leads to a relatively long vulnerable phase resulting in a predisposition to SHF [7,8,9]. The SHF is still regarded as a challenging fracture, particularly in cases of displacement or severe concomitant injuries [7]. Pediatric SHFs are predominantly classified according to the increased displacement and therefore the fracture severity by the Von Laer classification (type I–IV) in the European area [10,11]. Type I and stable type II fractures are treated conservatively. Type III and IV generally require surgical treatment. Among the several methods available, a common technique is the crossed insertion of Kirschner wires (K-wires). In the literature, the optimal technique for pin fixation is discussed. However, crossed K-wire fixation provides the highest stability and is suited for any kind of fracture pattern in contrast to lateral configurations, elastic stable intramedullary nailing or an external fixation [12,13,14,15]. The standard operation procedure is closed reduction and percutaneous pinning (CRPP) for fractures with an unstable fracture pattern including comminution, increased Bauman angle, obliquity over 20° and/or displacement as in fracture types III and IV [16,17,18,19,20]. In case of failed CRPP, soft tissue interposition, neurovascular entrapment, severe displacement, open fractures, limb ischemia (white pulseless hand) and neurological deficits, an open reduction (OR) with a lateral, medial, anterior or posterior approach is recommended [21,22,23]. Complications such as nerve lesions, cubitus varus or valgus deformity, impairment of range of motion (ROM) or pin track infections were seen in 6–18% after CRPP and OR [24,25,26,27,28]. One main issue of the crossed pinning technique is the risk of the ulnar nerve injury from the medial K-wire [29]. When open reduction seems necessary, still no reports on whether one of the approaches offered is superior are given [26,30,31,32,33,34]. Advantages and disadvantages are described for various approaches. The medial open approach is described as beneficial for preventing a rotational error by exposing the medial column and therefore reducing cubitus varus. Further, by an open medial approach identifying the ulnar nerve, the risk of iatrogenic ulnar nerve lesions can be reduced, which is the main risk stated in the literature [26,34,35]. The lateral approach offers the advantage of good exposure to posteromedially displaced fractures with a trapped metaphyseal spike [21,26,35,36]. The anterior approach is particularly recommended for injuries to the neurovascular bundle, but is not used very frequently as these injuries are not common [37,38,39]. The posterior or triceps-sparing approach also allows for the identification of the ulnar nerve and provides a good view of comminuted fractures. However, longer operation times, more ROM restrictions, cubitus varus deformities and ulnar nerve injuries have been reported [21,40,41].

The aim of this study was to describe the outcome of surgically treated pediatric supracondylar humerus fractures using crossed K-wires after closed or open reduction with a medial, lateral or bilateral approach.

## 2. Materials and Methods

This retrospective study was conducted with the approval of the Ethics Committee of the Medical University of Vienna (Code 1839/2020 on 8 September 2020) and according to the declaration of Helsinki and its latest amendments.

### 2.1. Inclusion/Exclusion of Patients

Children and adolescents aged from 0 to 18 years with surgically treated isolated SHF with crossed K-wires using closed or open reduction with a medial, lateral or bilateral approach at the University Clinic of Trauma Surgery at the Medical University of Vienna during an observation period from January 2000 to December 2019 were included. Exclusion involved incomplete data or treatment elsewhere.

### 2.2. Data Acquisition

Data collection was performed retrospectively from patient charts, saved at the data bank of the Medical University of Vienna called AKH information management (AKIM). Charts were detected in our hospital computer system by searching for the term “fractura supracondylaris humeri”, which generated all patients with this kind of fracture and further selected them by age to obtain all pediatric patients. The following parameters were collected: age, sex, side, time and mechanism of injury, treatment (surgical method/approach), radiological aspects, complications, follow-up and outcome (ROM, deformities, nerve lesions). All parameters presented were completely available for the entire cohort.

### 2.3. Classifications

Fractures were classified by the Von Laer classification [42]. A type I fracture is described as non-displaced, type II as displaced in one plane, type III as displaced in two planes and type IV as completely displaced in all three planes without contact between the fracture sites [42].

Complications were classified according to the classification system of Sink et al. [43]. This system describes surgical complications from grade I to V. Grade I complications do not require further treatment and therefore do not lead to deviation from routine FUP. The grades increase based on the severity of the complication and the treatment required, such as close monitoring (grade II), surgical revision (grade III), admission to intensive care (grade IV) or death (grade V).

The outcome was classified by the modified Flynn criteria [44]. These criteria determine excellent, good, fair and poor outcomes by cosmetic, functional and neurological deficits. Excellent, good and fair outcomes are declared to be satisfactory, while poor outcomes are unsatisfactory. A satisfactory result is achieved when the carrying angle changes by a maximum of 15 degrees, the range of motion changes by a maximum of 15 degrees and if nerve palsy persists for a maximum of 6 months after pin insertion. If one of these factors changes to a greater extent, the result is unsatisfactory.

### 2.4. Surgery and Postoperative Standard Protocol

**Figure 1:**
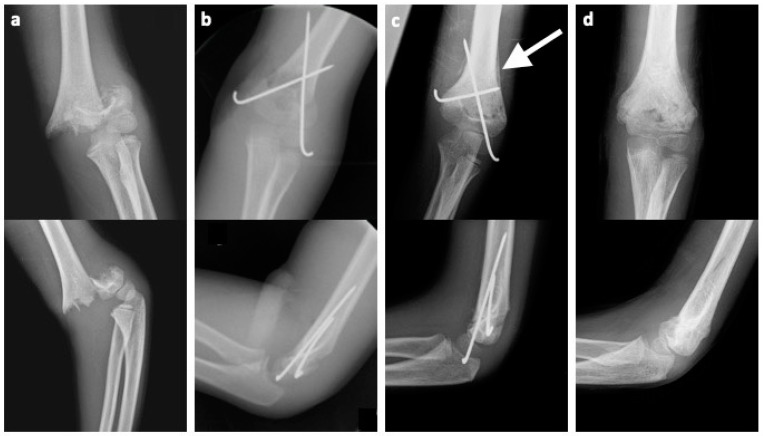
(**a**) Initial a.p. and lateral images of a Von Laer type IV supracondylar fracture in a seven-year-old girl. (**b**) Postoperative a.p. and lateral images illustrating an anatomical reduction and the crossed K-wire fixation. (**c**) Images after cast removal three weeks postoperatively (white arrow indicating callus formation). (**d**) Images of bony consolidation six weeks after initial surgery.

Surgery was performed according to our standard procedure with crossed K-wire fixation after CRPP and OR via the medial, lateral or bilateral approach. The lateral approach is made through an incision over the lateral supracondylar ridge. The fascia is then severed and the brachioradialis muscle can be guided anteriorly and the triceps muscle posteriorly, exposing the lateral column. For the medial approach, an incision is made over the medial epicondyle of the humerus. The dissection is now carried out in depth, whereby the ulnar nerve is visualized. If exposure and mobilization of the ulnar nerve are necessary, it is intended to restore anatomical conditions afterward. Changes are documented postoperatively. The dissection can now be continued to visualize the brachialis muscle and finally the medial aspect of the fracture. The postoperative standard protocol includes a cast application for 3 weeks in total. The first X-ray control and stitch removal were carried out after two weeks. The second X-ray control was after cast removal and permission for full ROM was given. Clinical evaluation was planned two weeks after cast removal and removal of K-wires usually was planned six weeks after surgery. Generally, all patients were rescheduled for clinical evaluation until full ROM was regained. A typical fracture case with operative treatment and radiological outcome is depicted in Figure 1.

### 2.5. Data Analysis

Descriptive analysis (mean ± standard deviation) was performed for the entire patient cohort. In order to provide an epidemiological overview, the parameters described above were included. For metric variables (age or FUP in days), mean values and standard deviation values and for categorial variables (fracture type, sex, type of treatment and treatment methods, outcome), frequencies and percentages were determined. Categorical data were tested with Fisher’s exact test. The Bonferroni–Holm Correction was performed for multiple tests. *p*-values were calculated for differences between total complication rates, complications requiring further treatment (grade II–V) and the outcome after OR and CRPP, and between complications demanding further treatment (grade II–V) and the outcome after a medial, lateral or bilateral approach. Statistical significance was set at a level of *p* < 0.05.

All statistical analyses were performed using Microsoft^®^ Excel macOS software (Version 16.79.2 Microsoft Corp., Redmond, WA, USA) and SPSS^®^ software (Version 29.0.1.0, SPSS Inc.: Chicago, IL, USA).

## 3. Results

### 3.1. Demographic Data

In total, the study population included 364 patients with a mean age of 5.23 ± 2.45 years (girls: 5.2 ± 2.4; boys: 5.3 ± 2.5) and consisted of 162 girls (44.5%) and 202 boys (55.5%). The left arm was fractured in 217 (59.6%) and the right one in 147 patients (40.4%). The classification by Von Laer showed 90 (24.7%) type-II, 118 (32.4%) type-III and 156 (42.9%) type-IV fractures.

The trauma mechanism led, in total, to 9 nerve affections (7× radial nerve; 1× median nerve; 1× ulnar nerve). One patient suffered from a simultaneous lesion of the median and radial nerve.

### 3.2. Treatment Methods and Approaches

CRPP was performed in 218 (59.9%) and OR in 146 (40.1%) fractures (Table 1a). The majority of type-II (73; 81.1%) and type-III (83; 70.3%) fractures could be treated by CRPP. In contrast, in 94 (60.3%) type-IV fractures, OR was performed.

The single medial approach was the most commonly used open approach (85/146; 58.2%) (Table 1b). The single lateral approach was chosen for eight fractures (5.5%). The bilateral approach was used for 53 (36.3%) fractures, and then predominantly in type-IV fractures (38/94; 40.4%).

### 3.3. Postoperative Complications According to Sink et al.’s Classification

Overall, 50 postoperative complications (33/50 grade I complications) occurred, whereas 17 (34%) needed further treatment (Table 2). One patient suffered from a simultaneous lesion of the ulnar and radial nerve, both of them were grade I complications and did not require further interventions. The total complication rate and the complications according to fracture types between closed (CRPP) and open (OR) reduction groups did not significantly differ (total: *p* = 0.484, II: *p* = 0.605; III: *p* = 0.190; IV: *p* = 0.573).

The 17/50 higher grade complications that required further treatment were seven (1.9%) grade II, including two ulnar and two radial nerve lesions, three K-wire skin perforations with associated infections and ten (2.8%) grade III complications, including nine ulnar nerve lesions and one deep wound infection. Surgical revision was undertaken in ten patients (2.8%).

In severely displaced fractures (type III and IV), the complications demanding further treatment were by trend higher after CRPP (type III: 7; 8.4%, type IV: 5; 8.1%) than after OR (type III: 1; 2.9%, type IV: 2; 2.1%) (*p* = 0.156) (Table 3a).

CRPP led to a total of 13 complications, 8 ulnar nerve lesions, 1 radial nerve lesion and 4 wound infections.

OR led to three complications after a single medial approach (3/85; 3.5%), one radial and two ulnar nerve lesions occurred. After a single lateral approach, one (1/8; 12.5%) lesion of the ulnar nerve was documented (Table 3b). Complications requiring further treatment did not differ significantly between the medial, lateral and bilateral approaches (*p* = 0.085).

### 3.4. Outcome According to Modified Flynn Criteria

Satisfactory outcome was shown by 359 (98.6%) patients. Five patients (1.4%) experienced limitations at the last FUP that resulted in an unsatisfactory outcome (Table 4).

Unsatisfactory results were documented as follows: one (an ulnar nerve lesion with extension deficit in the fifth finger) after CRPP in type II; two (an ulnar nerve lesion with hyposensitivity and a flexion restriction of 20°) after CRPP in type III; and two unsatisfactory results in type IV fractures. Further, the results were one after CRPP (extension deficit of 30° with simultaneous ulnar nerve lesion) and one after OR (extension deficit of 20 degrees).

Despite 50 surgical complications in 49 patients, a satisfactory outcome was achieved by 45 patients (91.8%). One grade I complication (K-wire skin perforation without infection) resulted in a poor outcome due to a flexion deficit of 20 degrees. Grade III complications led to three poor outcomes due to ulnar nerve lesions, including one with a motor deficit of a 30-degree extension deficit in the elbow.

In total, five poor results were documented. Four (1.8%) (one type-II, two type-III, one type-IV fracture) in the closed reduction group and only one (0.7%) after open reduction with a bilateral approach in a type IV fracture. No significant differences were present concerning the outcome between CRPP and OR (*p* = 0.334) and the different approaches (*p* = 0.418).

Fracture types II–IV show similar satisfactory outcome rates ranging from 98.3% to 98.9% after surgery (Table 5).

## 4. Discussion

In this study, an OR was predominantly used for severely displaced fractures. The more displaced and unstable the fracture, the higher the probability that an OR was required. The majority of type II and III fractures could be treated by CRPP, severely displaced fractures (Von Laer type IV) mostly needed an OR. For fractures requiring an OR, the complication rate was lower compared to CRPP with relatively similar clinical outcomes. Ducic et al. also showed in 93 fractures that an OR was predominantly used for severely displaced fractures (Gartland type III). Studies describing the treatment and outcome of surgically treated SHFs often include only severely displaced fractures in a patient population of less than 100 patients [26,27,41,45]. Karagoz et al. reveal a larger population of 198 patients but solely investigated OR maneuvers [35]. The complication rate of 13.7% may be slightly higher but is still comparable to the current literature ranging from 8.4% to 11.8% after OR [24,30,46]. However, it must be mentioned that the definition and classification of complications were handled differently in various studies [24,27,30,46]. Yavuz et al. also reported a complication rate of 10%, although 22 (11%) pin tract infections were not included. The relatively high rate of 13.7% in this present study can be explained by a relatively broad definition. However, a closer look reveals that 66% of these complications were grade I complications that did not require further treatment. Higher-grade complications occurred in 4.7% of all patients and only 2.8% needed surgical revision. Compared to the current literature and an actual revision rate of 4.3% to 10.3%, this percentage of 2.8% is relatively low [27,30]. The observation that the overall complication rate after OR was, by trend, lower than after CRPP also follows the literature. By trend, this was also observed by Abousaleh et al. in 60 Gartland type III fractures, which might suggest a benefit for OR in severely displaced fracture patterns; however, this would need further exploration [45].

In this study, a high number of satisfactory outcomes were achieved for both groups (OR and CRPP). CRPP tended to reflect slightly more poor outcomes of each fracture type than OR. Similar results for severely displaced SHFs type III and IV are described in the literature [26,27,45].

Further, this study investigated the outcome and complication rate of SHFs after open reduction according to the approach used (lateral, medial, bilateral).

The most frequently used approach was medial. The overall complications after OR requiring further treatment were low (three medial; one lateral). The type of complications occurring is also comparable to the current literature and mainly represents nerve lesions and superficial pin tract infections. Vascular damage and compartment syndrome did not appear and also rarely occur in the literature [24,25,39,47,48,49].

Also, no significant difference was noted when comparing the clinical outcome of these three approaches in achieving a fair equal number of excellent outcomes. Only one poor result of 38 patients was observed following a bilateral approach (extension deficit of 20 degrees). The high percentage of excellent outcomes and low numbers of complications indicate that open reduction independently of the three approaches (medial, lateral or bilateral) followed by crossed K-wire pinning can be considered a safe and effective method. In general, different studies compare some of the available approaches (anterior, lateral, medial, posterior), but, currently, no actual differences in outcome are described in the current literature [35,40,46,50,51]. However, various advantages and disadvantages are published for these different approaches.

The medial approach may prevent ulnar nerve lesions and cubitus varus deformity [21,34]. Abbott et al. recommend the lateral approach for lateral pinning only in order to avoid a possible nerve lesion in both CRPP and OR [24]. However, it must be mentioned that crossed K-wires are considered to represent the most stable configuration [13,14,15,42,52]. Furthermore, in a recent study, highly satisfactory outcomes could be reached with crossed K-wires. The anterior approach is favored to identify and repair nerve and vessel lesions [36,53]. Bombaci et al. claim that a perfect reduction is only possible with a posterior approach [40]. However, Sahin et al. showed that there was no difference between the outcome after the posterior and medial approaches, but surgery times were even longer with the posterior approach [54]. Furthermore, Kzlay et al. describe better functional results using a medial or a lateral approach than with the posterior approach [50].

Generally, the medial and lateral approaches are the most commonly described in the literature [26,30,34,35,55,56]. Current data on a bilateral approach are not yet available, with the exception of the present study. Among 53 bilateral approaches, not a single grade 2–5 complication and only 1 poor outcome was observed in this study. With an average satisfactory outcome rate of 52–100% in the current literature [26,30,35,41,46,55,56], the rate in this study is among the most satisfactory results for both, the bilateral approach (97.4%) and overall (98.6%). The bilateral approach, which has been used primarily for type 4 fractures, should therefore be considered especially in difficult cases of highly displaced and unstable fractures, as it does not lead to higher complication rates or worse outcomes than a unilateral approach.

Because of the overall similar results on the outcome but various general differences of approaches mentioned, it is still challenging to decide on the optimal approach for OR, if necessary. Summarizing, some recommendations can be formulated. In general, any anatomical structure must be considered. This includes neuro-vascular structures that may have already been injured by the fracture and may require primary revision/repair as the brachial artery and median nerve in the pink/pale pulseless hand. Further, the localization of the metaphyseal spike, which is often trapped in the soft tissue and prevents a successful reduction, should also be considered [21,57]. In case of extended swelling and therefore impaired ability to localize the ulnar nerve, a mini-open medial approach can be advised [58]. Generally, since the overall fair efficacy and safety of open approaches for pediatric supracondylar humerus fractures, a definite surgical approach (medial, lateral or bilateral) should be chosen according to the demands of the fracture pattern.

### Limitations

Finally, as a potential limitation, it should be noted that at our level 1 trauma center, all senior doctors treat pediatric fractures acutely when on call. This fact could have influenced the relatively high number of open reductions and perhaps also the type of approaches. However, postoperatively, all children are cared for in the pediatric outpatient clinic (a team of four senior physicians) according to a standard postoperative protocol, regardless of the closed or open reduction and the approach used. The fact that the first operation was performed by different doctors also seems to emphasize that the chosen approach generally does not influence the outcome of the open surgery. Of course, suggestions for a medial and lateral approach are included where the periosteal hinge is torn, which might facilitate reduction maneuvers [59]. Since it is possible to address the lateral column from a medial approach, and vice versa, cosmetic considerations and individual preferences must be taken into account as well. Therefore, the authors conclude that the fracture pattern did not determine the final approach alone, resulting in this variety of approaches. The exact number of surgeons who participated in the treatment in the specified period cannot be reproduced. The department has a constant staff of 60 to 70 surgeons and is subject to a relatively constant turnover of staff over the years. Considering that this study was conducted in one of the largest trauma units in the world and the largest pediatric trauma unit in the country, the results of this study can be considered representative. This is also reflected in the fact that the current study illustrates the largest patient cohort of operated SHFs (364; Von Laer type-II–IV fractures) in the current literature. Furthermore, this is the first study to describe the bilateral approach for OR with excellent results. The number of recent studies on severely displaced SHFs in the current literature reflects the continued relevance and importance of this topic and the need for further research [4,30,35,37,45,46,60].

## 5. Conclusions

Patients achieved excellent results after CRPP and OR using the crossed K-wire configuration, which can be considered a safe technique regardless of the open approach used. Data demonstrate that the higher the fracture dislocation, the higher the chance that OR is required to achieve adequate reduction and fixation. Data may suggest that severely displaced fractures may benefit from an open approach resulting in a trend of fewer complications than closed reduction, but this needs further exploration. However, it was shown that when open reduction was required, the chosen surgical approach did not influence the complication rate or clinical outcome. Therefore, when open reduction is needed, the required surgical approach (medial, lateral or bilateral) should be chosen according to the demands of the fracture pattern.

## Figures and Tables

**Table 1:** (**a**) The distribution of closed and open reduction in total and according to fracture types II–IV. (**b**) The distribution of surgical approaches used for open reduction according to fracture types II–IV.

(a)								
Method	Total		Von Laer Fracture Type					
	(n = 364)	%	II (n = 90)	%	III (n = 118)	%	IV (n = 156)	%
CRPP	218	59.9	73	81.1	83	70.3	62	39.7
OR	146	40.1	17	18.9	35	29.7	94	60.3
(**b**)								
**Open Reduction**	**Von Laer Fracture Type**							
	**Total** **n = 146/364** **(40.1%)**	**%**	**II n = 17/90** **(18.9%)**	**%**	**III** **n = 35/118** **(29.7%)**	**%**	**IV** **n = 94/156** **(60.3%)**	**%**
Medial	85	58.2	8	47.1	24	68.6	53	56.4
Lateral	8	5.5	3	17.6	2	5.7	3	3.2
Bilateral	53	36.3	6	35.3	9	25.7	38	40.4

**Table 2:** The distribution of the overall 50 postoperative complications (including the 33/50 grade I complications) classified by Sink et al. according to treatment methods (closed vs. open reduction) and fracture types II–IV.

		Total		Von Laer Fracture Type					
		n = 364	%	II n = 90	%	III n = 118	%	IV n = 156	%
	I	33	9	5	5.6	9	7.6	19	12.2
	II	7	1.9	-	-	3	2.5	4	2.6
Complication	III	10	2.8	2	2.2	5	4.2	3	1.9
Grade	IV	-	-	-	-	-	-	-	-
	V	-	-	-	-	-	-	-	-
	Total	50	13.7	7	7.8	17	14.4	26	16.7
Complication	No	187	85.8	67	91.8	69	83.1	51	82.3
CRPP	Yes	31	14.7	6	8.2	14	16.9	11	17.1
	Total	218	100	73	100	83	100	62	100
Complication	No	127	86.8	16	94.1	32	91.4	79	84
OR	Yes	19	13	1	5.9	3	8.6	15	16
	Total	146	100	17	100	35	100	94	100
Complication	No	314	86.3	83	92.2	101	85.6	130	83.3
Total	Yes	50	13.7	7	7.8	17	14.4	26	16.7
	Total	364	100	90	100	118	100	156	100

**Table 3:** (**a**) Complications (grade II and higher) requiring further treatment after closed (CRPP) or open (OR) reduction according to fracture types II–IV. (**b**) Complications requiring further treatment after open reduction using a medial, lateral or bilateral approach in type II–IV fractures.

(a)									
		Total		Von Laer Fracture Type					
**Approach**	Complication	n = 17/364 (4.7%)	%	II n = 17/90 (2.2%)	%	III n = 8/118 (6.8%)	%	IV n = 7/156 (4.5%)	%
CRPP	No	205	94	72	98.9	76	91.6	57	91.9
	Yes	13	6	1	1.1	7	8.4	5	8.1
OR	No	142	97.3	16	94.1	34	97.1	92	97.9
	Yes	4	2.7	1	5.9	1	2.9	2	2.1
(**b**)									
		**Total**		**Von Laer Fracture Type**					
**Approach**	**Complication**	**n = 4/146** **(2.7%)**	**%**	**II** **n = 1/17** **(5.9%)**	**%**	**III** **n = 1/35** **(2.9%)**	**%**	**IV** **n = 3/94** **(3.2%)**	**%**
Medial	No	82	96.5	7	87.5	23	95.8	52	98.1
	Yes	3	3.5	1	12.5	1	4.2	1	1.9
Lateral	No	7	87.5	3	100	2	100	2	66.7
	Yes	1	12.5	-	-	-	-	1	33.3
Bilateral	No	53	100	6	100	9	100	38	100

**Table 4:** Overall outcome of surgically treated fracture types (II–IV).

		Total		Von Laer Fracture Type					
Outcome		n = 364	%	II n = 90	%	III n = 118	%	IV n = 156	%
	Excellent	348	95.6	85	94.4	111	94.1	152	97.4
Satisfactory	Good	9	2.5	4	4.4	3	2.5	2	1.3
	Fair	2	0.5	-	-	2	1.7	-	-
Unsatisfactory	Poor	5	1.4	1	1.1	2	1.7	2	1.3

**Table 5:** The outcome of fracture types after CRPP od OR according to medial, lateral and bilateral approaches.

		CRPP		Open Reduction							
		n	%	Medial n	%	Lateral n	%	Bilateral n	%	Total n	%
	Total	73	100	8	100	3	100	6	100	17	100
	Excellent	69	94.5	8	100	2	66.7	6	100	16	94.1
II	Good	3	4.1	-	-	1	33.3	-	-	1	5.1
	Fair	-	-	-	-	-	-	-	-	-	-
	Poor	1	1.4	-	-	-	-	-	-	-	-
	Total	83	100	24	100	2	100	9	100	35	100
	Excellent	79	95.2	21	87.5	2	200	9	100	32	91.4
III	Good	1	1.2	2	8.3	-	-	-	-	2	5.7
	Fair	1	1.2	1	4.2	-	-	-	-	1	2.9
	Poor	2	2.4	-	-	-	-	-	-	-	-
	Total	62	100	53	100	3	100	38	100	94	100
	Excellent	61	98.4	53	100	3	100	35	92.1	91	96.8
IV	Good	-	-	-	-	-	-	2	3.5	2	2.2
	Fair	-	-	-	-	-	-	-	-	-	-
	Poor	1	1.6	-	-	-	-	1	2.6	1	1.1

## Data Availability

The datasets generated and/or analyzed in the current study are not publicly available due to data privacy but are available from the corresponding author upon reasonable request.
